# Moderate Mocha Coffee Consumption Is Associated with Higher Cognitive and Mood Status in a Non-Demented Elderly Population with Subcortical Ischemic Vascular Disease

**DOI:** 10.3390/nu13020536

**Published:** 2021-02-06

**Authors:** Francesco Fisicaro, Giuseppe Lanza, Manuela Pennisi, Carla Vagli, Mariagiovanna Cantone, Giovanni Pennisi, Raffaele Ferri, Rita Bella

**Affiliations:** 1 Department of Biomedical and Biotechnological Sciences, University of Catania, Via Santa Sofia 97, 95123 Catania, Italy; drfrancescofisicaro@gmail.com (F.F.); manuela.pennisi@unict.it (M.P.); 2 Department of Surgery and Medical-Surgery Specialties, University of Catania, Via Santa Sofia 78, 95123 Catania, Italy; pennigi@unict.it; 3 Department of Neurology IC, Oasi Research Institute-IRCCS, Via Conte Ruggero 78, 94018 Troina, Italy; rferri@oasi.en.it; 4 Department of Medical and Surgical Sciences and Advanced Technologies, University of Catania, Via Santa Sofia 87, 95123 Catania, Italy; carlavagli@gmail.com (C.V.); rbella@unict.it (R.B.); 5 Department of Neurology, Sant’Elia Hospital, ASP Caltanissetta, Via Luigi Russo 6, 93100 Caltanissetta, Italy; m.cantone@asp.cl.it

**Keywords:** coffee consumption, caffeine, cerebrovascular disease, executive dysfunction, geriatric depression, dose-response association

## Abstract

To date, interest in the role of coffee intake in the occurrence and course of age-related neurological and neuropsychiatric disorders has provided an inconclusive effect. Moreover, no study has evaluated mocha coffee consumption in subjects with mild vascular cognitive impairment and late-onset depression. We assessed the association between different quantities of mocha coffee intake over the last year and cognitive and mood performance in a homogeneous sample of 300 non-demented elderly Italian subjects with subcortical ischemic vascular disease. Mini Mental State Examination (MMSE), Stroop Colour-Word Interference Test (Stroop T), 17-items Hamilton Depression Rating Scalfe (HDRS), Activities of Daily Living (ADL), and Instrumental ADL were the outcome measures. MMSE, HDRS, and Stroop T were independently and significantly associated with coffee consumption, i.e., better scores with increasing intake. At the post-hoc analyses, it was found that the group with a moderate intake (two cups/day) had similar values compared to the heavy drinkers (≥three cups/day), with the exception of MMSE. Daily mocha coffee intake was associated with higher cognitive and mood status, with a significant dose-response association even with moderate consumption. This might have translational implications for the identification of modifiable factors for vascular dementia and geriatric depression.

## 1. Introduction

The progressive aging of the population has led to an increased rate of some age-related diseases, such as cognitive impairment and dementia, including Alzheimer’s disease (AD) and vascular dementia (VaD), as well as some late-onset neuropsychiatric disorders (i.e., geriatric depression). As an “umbrella term”, vascular cognitive impairment (VCI) encompasses a wide range of cognitive deficits due to neurovascular disorders, such as those resulting in subcortical ischemic vascular disease (SIVD), secondary to lacunar infarcts and vascular white matter lesions (WMLs) [1,2]. As such, VCI includes all types of vascular-related cognitive disorders, from the involvement of a single cognitive domain without dementia (called mild VCI) to a clear VaD [3].

In addition to cognitive impairment, behaviour and mood abnormalities are frequently found in association with ischemic lesions of the prefrontal-subcortical loops underlying mood and affecting control [1,2]. Indeed, SIVD is commonly associated with late-life depression, referred to as “vascular depression” [3], a clinical-radiological condition which has been found to be different from early-onset major depression [4,5]. Namely, the “vascular depression” hypothesis posits that cerebrovascular disease may predispose, precipitate, or perpetuate geriatric depression, thus shedding light on the complex relationships between late-life depression, WMLs, and cognition. Over time, the progression of WMLs and cognitive deficit also predicts a poor course of depression and drug-resistant response, both related to a worsening of the underlying SIVD [6]. Although still debated, a “disconnection hypothesis”, wherein focal vascular damage and WML location seem to play a crucial factor, contributes to the disease onset and course, as well as to clinical symptomatology and its severity. More recently, neuroinflammation and chronic hypoperfusion have also been linked to the vascular processes underlying cognitive dysfunction and late-life depression, eventually influencing their development and course [7,8,9,10].

Overall, both cognitive impairment and geriatric depression cause a significant impact on the social and healthcare-related burden worldwide [11,12,13]. Epidemiological evidence also supports the concept that modifiable vascular and lifestyle-related factors are related with the onset of cognitive impairment and movement disorders [14,15,16,17,18,19,20]. To date, an early identification and prompt management of cardio- and cerebrovascular risk factors are the only effective measures to prevent dementia and other types of cognitive disorders, such as Mild Cognitive Impairment (MCI) and VCI [21,22]. Therefore, a comprehensive description and quantification of these factors is the preliminary step towards the elucidation of the causes and mechanisms underlying the onset and progression of dementia, as well as the design of new disease-modifying strategies [23,24,25,26,27,28,29,30,31].

Some dietary components of the Mediterranean diet have been traditionally considered as preventing factors of cardiovascular diseases (including stroke), and some age-related cognitive disorders (such as AD and VaD) [32,33,34,35,36]. Recently, there has been increasing interest in the exploration of the role of coffee intake in some neurological and neuropsychiatric disorders, although large epidemiological investigations are still inconclusive in terms of a protective role of caffeine in the risk of cognitive disorders. Indeed, while many studies have found a protective role of coffee in cognitive impairment [37], an extensive neuropsychological evaluation was not always performed, and the association was not found, or at least not for all, cognitive domains [37]. Similarly, there is still no consensus regarding a dose-response effect on cognition or mood [37,38,39,40,41].

Regarding the risk of depression, a systematic review and dose-response meta-analysis of observational studies on coffee, tea, and caffeine [42], including 23 datasets accounting for a total of 8146 individuals with depression, found that, compared to those with lower coffee consumption, the higher intakes had a pooled relative risk of depression of 0.76 (95% confidence interval 0.64, 0.91). The dose-response effect suggested a nonlinear “J-shaped” relation between coffee consumption and risk of depression, with a peak of effect for 400 mL/day, suggesting a protective role of coffee in the risk of depression [42]. Another meta-analysis of observational studies confirmed that coffee and caffeine consumption were significantly associated with a decreased risk of depression [43].

The roasted seeds of *Coffea* sp. are used for coffee extraction. The composition of a coffee beverage basically depends on species, roast, and preparation methods [44,45,46], which vary according to geographic and cultural factors, all affecting the chemical profile. For instance, in Italian coffee shops, the espresso method, in which hot water at high pressure is passed through about 8 g of finely-ground coffee powder producing a serving of 30 mL, is basically the rule. Another common preparation of coffee in Italy, particularly at home and in the Southern regions, is through a mocha, i.e., an aluminium or stainless-steel machine, in which hot water (approx. 70 °C) is forced up through the coffee in the pot top [47].

Methods of preparation influence diterpenes and caffeine concentration. Cafestol and kahweol content per cup is approximately 7.2 mg in boiled coffee, 2.3 mg in mocha coffee, 1.0 mg in espresso coffee, but only 0.02 mg in filtered coffee [48]. In Italian-made coffee, the contact time of water with the solid coffee is very short, thus accounting for the lower concentration of lipids than boiled coffee, although it is substantially higher than paper-filtered coffee [48]. However, albeit the concentration of diterpene in Italian-made coffee is considerable [48], the size of the cup is much smaller than in other European countries. On these bases, differences in the effects of mocha coffee are expected.

As drank daily by millions of persons worldwide and due to the caffeine content, coffee is the most common psychophysical stimulating agent, whose consumption is known to result in heightened alertness and arousal, as well as in better mental performance [49]. Studies in male rats also indicate that long-lasting caffeine intake might prevent cognitive deficits [50]. The main proposed mechanisms, among others, are based on the finding that caffeine, as an antioxidant compound, can limit oxidative stress [51] and exert neuronal protection against the disruption of the blood–brain barrier (BBB) [52].

In addition to its short-term effects, a systematic review of human studies has assessed the chronically assumed impact of coffee on cerebral functions, providing hints on the protective role in cognitive disorders till dementia, with a higher effect in females than in males [53]. However, the association was not observed for every cognitive domain and a clear dose-response association is still lacking [37]. It has also been shown that the risk of depression decreased with increasing caffeinated coffee consumption in women [40].

Similarly, the effects of coffee on VCI and geriatric depression remain basically unexplored, with previous studies frequently producing contradictory results. In a previous study [41], the association of coffee intake in midlife with the risk of dementia, its neuropathological correlates, and cognitive impairment was examined among 3494 men, including 418 decedents who underwent brain autopsy. Although dementia was diagnosed in 226 men (including 118 AD and 80 VaD) and cognitive impairment in 347, there was no association between coffee intake and the risk of cognitive impairment, overall dementia, AD, VaD, or moderate/high levels of neuropathologic lesions. However, men in the highest quartile of caffeine intake were less likely to have any lesion type at autopsy than men in the lowest quartile [41]. A few years later, the association between coffee intake and silent brain infarcts in magnetic resonance imaging (MRI) in 242 middle-age healthy individuals demonstrated that brain lesions were observed less frequently in those consuming three cups or more of coffee per day [54].

Lastly, a recent umbrella review evaluating current evidence of the relation between coffee consumption and human health suggested that there might be a possible association between coffee intake and decreased risk of AD [55]. However, the evidence was concluded from studies investigating the consumption of regular coffee, while data on mocha are lacking.

Based on these considerations, in the present study we aimed to estimate the association between mocha coffee consumption and cognitive and mood status in a homogeneous population of non-demented elderly Italian subjects with SIVD. We hypothesized that daily coffee intake would positively influence cognitive performance and mood status, with a dose-response association.

## 2. Materials and Methods

### 2.1. Participants

In this cross-sectional study, a population of 300 individuals was consecutively enrolled from the Cerebrovascular Diseases Centre of the “Azienda Ospedaliera Universitaria Policlinico Gaspare Rodolico-San Marco” of Catania, Italy. All subjects were referred to an expert neurologist (R.B.) because of non-specific cognitive deficits, with or without concomitant mood complaints.

An age ≥ 65 years, a Mini Mental State Examination score (MMSE) ≥ 24, and brain MRI-based evidence of lacunar state or ischemic WMLs were the inclusion criteria. Coffee consumption over the last year was focused to mocha, as it represents the most common preparation of coffee consumed by elderly people in Southern Italy. Exclusion criteria were: overt dementia, consumption of other coffee preparations different from mocha, any medical condition or drug affecting cognitive functions and/or mood status, alcohol or drug abuse, and any contraindication to MRI. Namely, 46 subjects mainly drinking espresso coffee or with a mixed coffee consumption (i.e., espresso and mocha) were excluded.

Signed informed consent was provided by all individuals before participation in the study, which was approved by the Ethics Committee of the “Azienda Ospedaliera Universitaria Policlinico Gaspare Rodolico-San Marco” of Catania, Italy (approval code 292/prot. n. 871) and performed according to the 1964 Declaration of Helsinki and its subsequent amendments.

### 2.2. Assessment

All participants underwent a detailed clinical and demographic history and a full neurological examination. In particular, information about past medical history, coffee consumption, smoking habit, and drugs taken was collected and confirmed by the subjects and their relatives/caregivers.

High blood pressure was considered as drug therapy intake or a measured systolic blood pressure ≥ 140 mmHg and/or diastolic blood pressure ≥ 90 mmHg. Hyperlipidemia and diabetes were assessed by a recent laboratory test or self-reported diagnosis and drug treatment. Given the lack of a validated food-frequency questionnaire on mocha coffee consumption, information on coffee data was collected via a diet record. The intake of coffee was classified as: <1 cup/day (non-drinkers or occasional consumers), 1 cup/day (light consumers), 2 cups/day (moderate consumers), and ≥3 cups/day (heavy consumers), as adapted from a recently published study [56].

Neuropsychological evaluation, performed by a trained neurologist (F.F.), who was blind with respect to coffee consumption, included: a screening test of cognitive functions, i.e., the Mini Mental State Examination (MMSE) [57]; a test evaluating executive functions and other frontal lobe abilities, i.e., the Stroop Colour-Word Interference Test (Stroop T) [58]; an assessment of depressive symptoms through a standardized questionnaire, i.e., the 17-item Hamilton Depression Rating Scale (HDRS) [59]; a scoring of the functional status according to the Activities of Daily Living (ADL) and the Instrumental ADL (IADL) [60].

As known, frontal lobe abilities are typically involved in this population, who may manifest attention deficit and executive dysfunction early, which causes impairment in complex information use, strategy formulation, and emotional-behavioural control [61]. Compared with AD, subjects with VCI also show a less pronounced impairment of episodic memory, but more depressive symptoms and greater variability in the disease course [6]. Moreover, SIVD due to small vessel disease contributes to the deterioration of psychomotor speed, global cognitive function, impaired executive control, and loss of activities of daily living, eventually leading to a considerable risk of dementia [62]. Cognitive impairment is also common in vascular depression, particularly executive dysfunction, a finding which is also predictive of poor antidepressant response [63].

All subjects underwent brain MRI, which was carried out with a 1.5 Tesla General Electric machine (General Electric Healthcare, Milwaukee, WI, USA). The neuroimaging exam included T1-, T2-, fluid-attenuated inversion recovery (FLAIR), and proton density-weighted scans (slice thickness 5 mm, slice gap 0.5 mm). The Fazekas visual scale was used for grading WMLs in all participants: 0 = lack of foci; 1 = punctuate foci; 2 = initially confluent foci; 3 = large confluent foci [64]. Lacunar lesions had to be of 3–20 mm, multiple (>5) cavitated lesions, with T2-weighted or FLAIR hyperintensity in the deep grey matter, corona radiata, and internal capsule. Foci less than 2 mm were considered perivascular spaces, with the exception of the anterior commissure, where larger perivascular spaces can be present. Another author (G.L.), blind to the clinical features and coffee intake, independently reviewed all the scans.

### 2.3. Statistical Analysis

Subjects’ characteristics at baseline, based on the subgroups of coffee consumption, were handled as means and standard deviations (continuous variables) or frequencies (categorical variables). Comparison of continuous variables obtained from the four groups of subjects was first carried out by means of the one-way ANOVA, while the comparison of categorical variables obtained from the same groups of subjects was performed by means of the chi-square test.

Then, based on results obtained from the above analyses, we checked for any simultaneous association of coffee consumption, as well as age, sex, education, and smoking (independent factors/predictors), on the scores obtained at the MMSE, HDRS, and Stroop T, separately (dependent variables) by means of the General Regression Models of the software STATISTICA v.6 StatSoft Inc., Tulsa, OK, USA (employed for all the statistical tests in this study). This module allows us to build models for design with categorical predictor variables, as well as with continuous predictor variables. For each dependent variable, the statistical significance of the association of coffee consumption was obtained by taking into account the effect of the other independent factors. Post-hoc comparisons between the different pairs of groups were then carried-out, for significant comparisons at the previous analysis, with both the Fisher Least Significant Difference (LSD) and the Tukey Honestly Significant Difference (HSD) tests.

Lastly, we analysed the association between MMSE, HDRS, or Stroop T and coffee consumption by calculating the Pearson’s correlation coefficient and, following Cohen’s [65] indications, we considered a correlation coefficient 0.10, 0.30, and 0.50 as corresponding to small, medium, and large sizes, respectively.

## 3. Results

The comparison between the different parameters in the four groups of subjects, established on the basis of their daily coffee consumption, is reported in Table 1 and Table 2. In Table 1, showing the comparison of continuous variables, a significant difference was found not only in most of the outcome measures (i.e., MMSE, HDRS, and Stroop T), but also in age and education, which might be viewed as factors potentially affecting the results. This was also observed for the comparison of some of the categorical variables (Table 2), such as sex and smoking.

**Figure 1:**
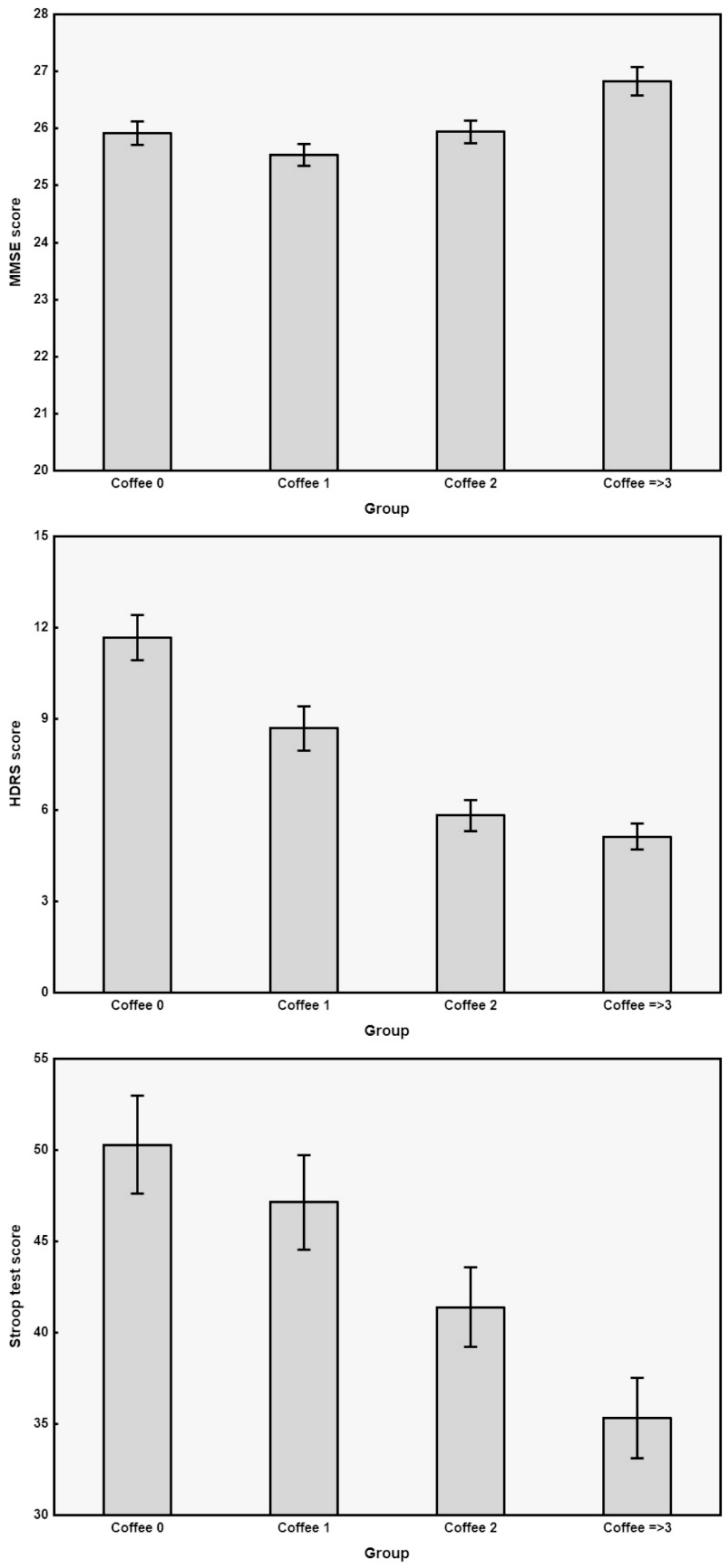
Bar graphs of continuous outcome variables obtained from the four groups of subjects and showing significant differences in Table 1. Data are shown as means (columns) and standard error (wishers). Coffee 0 = non-coffee drinkers; Coffee 1 = consumers of 1 cup daily; Coffee 2 = consumers of 2 cups daily; Coffee ≥ 3 = consumers of 3 or more cups daily; MMSE = Mini Mental State Examination; HDRS = 17-item Hamilton Depression Rating Scale; Stroop T = Stroop Colour-Word Interference Test.

In order to disentangle the association of coffee intake from that of other parameters that were found to be significantly different in the subject groups, we built an analysis design by using the above-mentioned General Regression Models module, using age, sex, education, smoking and coffee consumption as independent predictors, and MMSE, HDRS, Stroop T, ADL, and IADL as dependent variables. Accordingly, we were able to assess that MMSE, HDRS, and Stroop T scores were significantly and independently different in the three coffee consumption subgroups, with a generally lower severity of HDRS with increasing coffee intake (F = 22.669, *p* < 0.000001), as well as with better MMSE (F = 4.105, *p* < 0.0071) and Stroop T (F = 4.806, *p* < 0.0028). The results of MMSE, HDRS, and Stroop T are graphically displayed in Figure 1 as bar graphs of continuous outcome variables obtained from the four groups of subjects (i.e., non-drinkers and drinkers of different cups of mocha coffee per day).

Post-hoc analyses of the differences between the various pairs of groups showed that, for MMSE, the group with highest coffee daily consumption (≥three cups/day) had significantly higher values than the other groups (with both the Fisher LSD and the Tukey HSD tests). Additionally, regarding HDRS, the coffee ≥three group had the smallest scores which were, however, not significantly different from those of the coffee two group, and both groups (i.e., coffee ≥three and coffee two) had significantly lower scores than the other groups. Similarly, for the Stroop T, in which a significant decrease was observed with increasing coffee consumption, the post-hoc comparisons revealed no difference between coffee two and ≥three groups.

The simple (not adjusted) regression analysis between HDRS and individual daily coffee consumption in the whole group of subjects showed a correlation coefficient that can be considered to be medium-to-large, according to Cohen [65] (−0.386; *p* < 0.000001). The same analysis yielded a correlation coefficient of 0.193 (*p* < 0.0008, small-to-medium size) between coffee consumption and MMSE, and of 0.256 (*p* < 0.000001, close to medium size) between daily coffee consumption and Stroop T.

## 4. Discussion

### 4.1. Main Findings

This is the first study focused on the association between a specific coffee preparation method (i.e., mocha) and cognitive impairment and late-life depression in a homogeneous population of non-demented elderly subjects with SIVD. Most of the previous studies, indeed, explored the effects of boiled or filtered coffee, which are very popular in other European countries and in the USA [66]. An association between coffee consumption and cognitive and mood performance in VCI subjects, along with a dose-response association already observed with a moderate intake (two cups/day), without difference for heavier drinkers (with the exception of the MMSE results), are the main findings of this study.

A number of longitudinal and cross-sectional population-based studies hypothesized a protective role of coffee in cognitive impairment [37], although not all performed an extensive neuropsychological evaluation and, among them, the association was not found in all cognitive domains. In general, with increasing quantity of coffee, a positive dose-response effect was observed in cognitive batteries evaluating verbal memory, reaction time, and visuo-spatial skills [38], with higher lifetime coffee intake associated with higher scores [39]. On the other hand, in a cohort of elderly subjects, a significant association between coffee intake and overall cognitive and mnesic performance was found, although, after adjustment for age, intelligence quotient, and social status, this association became not significant [67]. In our population, we found a significant difference in age and education among the four groups, but the General Regression Model showed that the associations between mocha coffee intake and both cognition and mood were independent from the socio-demographic variables.

Other longitudinal studies found a negative association between coffee intake and cognitive disorder in a sample of healthy elderly men from different European countries [68], especially in women [69,70,71]. In particular, higher coffee consumption was associated with a moderately better maintenance of cognitive status over five years in older females with vascular diseases [72]. Such gender-related association was not evident in the present population of VCI subjects, as well as the association with vascular burden. Even for these aspects, previous evidence in this type of population is scarce and not tailored to the coffee preparation method. Regarding the gender-related effect, an earlier prospective study [73] on 455 participants (314 men) found that the incidence of small vessel disease was lower in male drinkers than male non-drinkers and occasional drinkers, whereas the incidence of WMLs was lower in female drinkers than female non-drinkers or occasional drinkers. In the multivariate analyses including age, sex, smoking status, body-mass index, and coffee consumption, the incidence of microbleeds was significantly lower in male drinkers compared to non-drinkers [73]. Regarding the vascular burden, a recent study [74] assessing the impact of some dietary intake (including coffee) on cognitive outcome and MRI parameters in old age demonstrated an inverse association between coffee consumption and cognitive performance. Moreover, moderate-to-heavy coffee drinking was associated with better white matter preservation and cerebral blood flow in cognitively stable elders [74].

It should be reported that some prospective reports did not observe any association between coffee intake and cognitive disorders [41,75,76,77]. Among 418 subjects who underwent brain autopsy, there was no association between coffee consumption and the risk of cognitive impairment, VaD, AD, or moderate/severe neuropathological lesions [41]. In 2015, a meta-analysis reached the conclusion that coffee or tea consumption was not associated with a risk of cognitive decline [78]. Nonetheless, the majority of systematic reviews and meta-analyses seem to converge on a positive effect of caffeine on cognitive decline [79,80,81], with a greater impact in females than in males [53], along with the minimum risk at a daily intake of one–two cups of coffee [82]. Similarly, two meta-analyses showed that coffee intake was significantly associated with a reduced risk of depression [42,43]. Accordingly, we found that subjects with the highest daily coffee consumption had significantly better MMSE compared to the other groups.

Finally, little is known about coffee and depression in older adults, as well as a dose-response association. According to our data, the positive association with HDRS and Stroop T seems to be dose-dependent, with a significant association reached with a moderate intake (two cups/day) and not further increasing with higher intake. In this context, it has been previously demonstrated that drinking coffee or tea was associated with lower risk for depression in older adults [83], whereas in another study on middle-age participants, those who drank at least four cups of coffee per day showed a significantly lower risk of depression than participants drinking ≤one cup, although an inverse linear dose-response association was not observed [84]. Finally, a very recent multicentre cross-sectional study on 1992 elderly Japanese women [85] found that coffee intake was associated with a lower prevalence of depressive symptoms in a fully adjusted model. Caffeine intake was also associated with depressive symptoms, although the association was not statistically significant, suggesting that the association might also be related to other substances in coffee or other factors related to coffee intake [85].

A recent study in Italy has evaluated the association between modification or constant habits in coffee intake and the occurrence of MCI in a sample of elderly subjects followed-up for 3.5 years [86]. This study found that individuals who usually drink a moderate daily quantity of coffee (one–two cups) exhibited a lower rate of MCI compared to non-drinkers or occasional consumers. Those who increased coffee consumption (>one cup) shower a higher incidence rate of MCI than subjects with constant habits (up to +/− one cup) or those without consumption. Additionally, no significant association between higher quantity of coffee intake (>two cups) and the incidence of MCI compared with non-drinkers or occasional consumers was observed [86]. Conversely, heavy and prolonged coffee consumption is associated with the risk of WMLs in later life, likely through an increase in arterial stiffness, vascular resistance, and cerebral vasoconstriction. This may result in reduced cerebral blood flow and eventually lead to an increased rate and severity of vascular lesions [87].

Lastly, tobacco smoking reduces the half-life of caffeine [88], a finding that might contribute to a weaker protective effect of coffee in smokers. Therefore, given that smokers are often coffee consumers, a weaker protective role is likely to take place in males, with the rate of smokers being higher in males than in females [89,90,91]. Although in our sample we found a significant difference in smoking habits among the groups, there was no significant smoking-related independent association with the outcome measures.

### 4.2. Limitations

These findings need to be considered in light of some limitations. Firstly, due to its cross-sectional design, this study is prone to several biases, including that of drinking habit as an effect of cognitive status rather than causally related [53]. Additionally, population-based studies may suffer from item response bias in the measures of cognition. Therefore, we cannot establish a causal association between these factors. Similarly, although a dose-response association is likely to occur, a causal relationship cannot be established. Future investigations, along with multidimensional follow-up, will verify the possibility of a protective role of mocha coffee consumption in the measures of interest, as well as a dose-response effect.

Secondly, not all cognitive domains were explored in this study, although we focused on those domains typically and early affected in subjects with VCI.

The self-reported coffee drinking habits may represent an additional limitation, although measurement errors are often inevitable for dietary exposures, especially in older individuals or in some with cognitive impairment, who may hardly have a reliable record of their usual consumption.

Fourthly, although the results were adjusted for possible confounding factors, we cannot entirely exclude the potential association with unmeasured confounders. For instance, coffee consumption may be related to other lifestyle and social factors that may account for part of the associations observed. This also holds true for medications assumed for the control of co-morbid conditions.

Lastly, although we hypothesized that caffeine would have contributed to the results, the potential contribution of other compounds contained in coffee (for instance, flavonoids) cannot be excluded and deserves further investigation.

## 5. Conclusions

Moderate mocha coffee consumption was associated with higher cognition and mood status in non-demented elderly subjects with VCI. If confirmed in larger populations, these findings may be implicated in the delay or even prevention of some age-related cognitive disorders and late-life mood disturbances. Prospective studies will elucidate the potential protective effect of different methods of coffee preparation both in aged healthy individuals and in patients with cognitive decline and geriatric depression, thus contributing to disentangling the exciting relationships between coffee intake and the aging brain.

## Figures and Tables

**Table 1:** Comparison of continuous variables obtained from the four groups of subjects.

	Coffee 0 cup/day (*n* = 73)		Coffee 1 cup/day (*n* = 69)		Coffee 2 cups/day (*n* = 87)		Coffee ≥ 3 cups/day (*n* = 71)		ANOVA	
	Mean	SD	Mean	SD	Mean	SD	Mean	SD	F_3,296_	*p*<
Age, years	73.9	6.2	72.9	5.7	73.5	6.4	70.9	5.6	3.623	0.014
Education, years	6.5	3.7	6.9	4.0	7.5	4.0	8.2	3.9	2.635	0.05
MMSE	25.9	1.8	25.5	1.6	25.9	1.9	26.8	2.1	6.212	0.00042
ADL	5.6	0.7	5.5	0.7	5.6	0.6	5.8	0.6	2.112	NS
IADL	7.2	1.3	6.8	1.5	7.1	1.2	7.3	1.2	1.814	NS
HDRS	11.7	6.4	8.7	6.0	5.8	4.8	5.1	3.6	23.790	0.000001
Stroop T	50.3	22.9	47.1	21.6	41.4	20.4	35.3	18.6	7.182	0.00011

Legend: SD = standard deviation; MMSE = Mini Mental State Examination; ADL = Activities of Daily Living; IADL = Instrumental Activities of Daily Living; HDRS = 17-item Hamilton Depression Rating Scale; Stroop T = Stroop Colour-Word Interference Test; NS = not significant.

**Table 2:** Comparison of categorical variables obtained from the four groups of subjects.

	Coffee 0 cup/day (*n* = 73)	Coffee 1 cup/day (*n* = 69)	Coffee 2 cups/day (*n* = 87)	Coffee ≥ 3 cups/day (*n* = 71)	Chi-Square	*p*<
Sex, male/female	28/45	32/37	48/39	48/23	13.60	0.004
Hypertension, yes/no	60/13	50/19	70/17	59/12	3.05	NS
Diabetes, yes/no	15/58	20/49	24/63	26/45	4.62	NS
Hypercholesterolemia, yes/no	22/51	19/50	29/58	28/43	2.54	NS
Coronary artery disease, yes/no	10/63	13/56	9/78	14/57	3.52	NS
Tobacco smoking, yes/no/ex	7/54/12	13/47/9	15/57/15	26/37/8	17.90	0.006
Atrial fibrillation, yes/no	11/62	7/62	8/79	9/62	1.56	NS
Neurologic signs, yes/no	29/44	30/39	36/51	32/39	0.49	NS
Family history, yes/no	8/65	13/56	8/79	8/63	3.70	NS
History of depression, yes/no	15/58	17/52	29/67	16/55	2.40	NS
MRI, lacunar/Fazekas 1/2/3	13/15/29/16	8/22/24/15	15/21/32/19	12/21/24/14	3.81	NS

Legend: ex = former smokers; MRI = magnetic resonance imaging; Fazekas 1/2/3 = white matter lesion severity based on the Fazekas visual scale: 0 = lack of foci; 1 = punctuate foci; 2 = initially confluent foci; 3 = large confluent foci; NS = not significant.

## Data Availability

The data that support the findings of this study are openly available in “Mendeley Data” at https://data.mendeley.com/datasets/vnzpzcw224/1.
